# The Delivery of Health Services and Telehealth to Provide Aged Care During COVID‐19 in the Kimberley Region of Western Australia

**DOI:** 10.1155/nrp/5493017

**Published:** 2026-07-08

**Authors:** Amanda Timler, Jane Gaspar, Karen Clark-Burg, Caroline Bulsara, Maria Morgan, Cathryn Josif, Erina Myers

**Affiliations:** ^1^ Institute for Health Research, University of Notre Dame Australia, Fremantle, Western Australia, Australia, nd.edu.au; ^2^ School of Health Sciences, University of Notre Dame Australia, Fremantle, Western Australia, Australia, nd.edu.au; ^3^ Child and Adolescent Health Service, Department of Health Western Australia, Nedlands, Western Australia, Australia; ^4^ School of Nursing and Midwifery, University of Notre Dame Australia, Fremantle, Western Australia, Australia, nd.edu.au; ^5^ Majarlin Kimberley Centre for Remote Health, Broome, Western Australia, Australia

**Keywords:** aged care services, Kimberley, remote, telehealth

## Abstract

**Objective:**

Objective: The aim of this project was to understand aged care service providers’ (ACSP) experiences of providing care during the COVID‐19 travel restriction period.

**Setting:**

Setting: Aged care facilities and community‐based settings within the Kimberly region of Western Australia.

**Participants:**

Participants: A total of 16 participants from 11 ACSPs took part in the project.

**Design:**

Design: A convergent mixed‐method design was employed for this project. A Project Advisory Committee (PAC) helped to inform the direction and robustness of this project. An online survey and semistructured interviews were used to collect quantitative and qualitative data.

**Results:**

Results: The key themes that emerged from this project included ‘*rapid changes to services,’ ‘importance of community*’ and ‘*long-term impact on the older people.’* These themes were impacted by barriers (access to services, resourcing, and equipment, staffing shortages and burnout, and government mandates, reporting and funding) and ameliorated by enablers (adapting existing services, supply of equipment and resources, staff training and support and changes to strategies and planning procedures).

**Conclusion:**

Conclusion: This project was informed by the PAC to ensure real‐world implications were considered. In health emergencies, additional resources such as improved infrastructure and telehealth, providing cultural safety training, avoiding a ‘one fits all approach’ for ACSP, and funding for staff retention could be considered.

**Summary**
• What is already known on this subject?
◦ Existing research has found additional challenges, such as limited access and resources.
◦ Staffing shortages often arise for nurses who provide aged care in rural and remote areas.
• What this paper adds?
◦ The additional stress of a global pandemic and the impact this had on nursing aged care among the elderly has not been previously considered.
◦ The type and frequency of service provision were adjusted to accommodate for lockdown restrictions and lead to longer term health impacts for the elderly.
◦ Aged care provided in rural and remote areas in Australia during a global pandemic has not been extensively studied.

## 1. Introduction

In March 2020, the World Health Organization (WHO) declared nCoV‐19, coronavirus disease (COVID‐19) a global pandemic [1]. In response to the evolving health crisis, Australian federal and state governments began to enforce border controls to limit the spread of infection. On 22 March 2020, the Western Australian (WA) government implemented arrival requirements for travel into WA from other states and territories and—to further protect those living in the Kimberley and remote Aboriginal communities—enforced additional COVID‐19 travel restrictions into the region from 26 March until 4 June 2020 [2] (hereinafter, the COVID‐19 restriction period) [3]. As occurred globally, these constraints impacted the flow of resources and services to communities, including aged care services^1^ [4–6].

Soon after declaring COVID‐19 a pandemic, the WHO acknowledged older people as amongst the most vulnerable and at highest risk from the virus and emphasised the importance of protecting older people living alone in the community and supporting those who provide aged care services [7]. Emerging pandemic studies indicate an escalation of stress and anxiety levels amongst aged care service providers (ACSPs) linked to their fear of spreading infection, inability to provide care to clients or ameliorate their social isolation [8]. Older people are already at an increased risk of social isolation, which has been exacerbated by COVID‐19 restrictions [9]. This is especially true for remote communities where staffing and resourcing shortages are already problematic, as is the case in the Kimberley [10].

The location of populations in very remote areas creates a challenge for care provision across the Kimberley region. Extreme weather events often render roads into remote communities impassable during the tropical wet season. Service delivery is further hindered due to socioeconomic disadvantage, high staff turnover, high burden of disease and ineffective funding arrangements that characterise the region [11, 12]. Add the complexities of a pandemic, and stretched services further struggle to meet community and staff needs. Early outcome data and research have shown that the unexpectedness of the COVID‐19 pandemic resulted in the need for timely and planned strategies to keep Kimberley communities safe [13, 14].

Telehealth^2^ technology enables health professionals to reach clients who are, otherwise, difficult to access [15, 16]. Telehealth and technology platforms for virtual health care facilitation gained prominence during the pandemic as patients were isolated for reasons such as border restrictions, social distancing and/or quarantining requirements [17]. The onset of COVID‐19 has been described as bringing about a ‘…new virtual medical world order…’ in which telehealth has enabled provision of care at a physical distance that maintains patient safety and accessibility [18, 19]. Prior to the pandemic, 12 percent of WA outpatient appointments were held via telehealth to patients in the Kimberley region [20]. It was an assumption of this project that the application of telehealth methods for the provision of aged care increased and was enhanced during the COVID‐19 restriction period.

## 2. Aims

This project encompassed five main aims: (1) describe services delivered to residential aged care facility (RACF) dwelling adults and older community members in the Kimberley during the COVID‐19 restriction period; (2) investigate the application of telehealth services and how these were adapted to deliver care to older populations during the pandemic; (3) consider barriers and enablers to providing aged care services during the pandemic in both residential and community‐based services; (4) explore experiences of nurses, allied health and other relevant direct care staff during the restriction period; and (5) explore strategies used by nurses, allied health and other stakeholders during the pandemic to ameliorate effects of isolation and travel restrictions for themselves and persons for whom they cared.

## 3. Materials and Methods

### 3.1. Setting

The project was undertaken in the remote and sparsely populated Kimberley region of northwest WA. The Kimberley encompasses an area of more than 420,000 square kilometres and comprises one‐sixth of Australia’s land mass [21]. At the 2016 Census, the Kimberley had an estimated resident population of 36,392 people, two per cent of whom were 75 years and over, and the number of people 70 years and over was projected to increase dramatically (by 111%) between 2017 and 2026 [22].

According to the Accessibility/Remoteness Index of Australia (ARIA), most of the Kimberley region (97%) is classified as ‘Very Remote.’ The remaining three per cent comprises the towns of Broome and Kununurra, which are classified as ‘Remote’ [22]. Other population areas include the towns of Derby, Fitzroy Crossing, Halls Creek and Wyndham, around 100 pastoral stations and 150 discrete Aboriginal communities, within which an estimated 50% of the Aboriginal population who live in the Kimberley region reside [13, 22].

### 3.2. Study Design

Given the range of providers and workforce in the Kimberley, a case study methodological approach was deemed most appropriate and rigorous method to address the research question. A case study is a multiperspective, in‐depth exploration of the complexity of a system in a ‘real life’ context to generate thorough understanding and detailed knowledge of said system [23]. The case study design incorporated a mixed‐methods approach that facilitated the exploration of experiences and perspectives of ACSPs, wherein the case was the aged care delivery sector operating within the Kimberley region. By exploring staff perspectives at all levels, the case‐study method provides a holistic picture of enablers and barriers to service delivery during the COVID‐19 restriction period. The line of inquiry focused only on the COVID‐19 restriction period although comparisons were made with delivery of services before the onset of COVID‐19.

A convergent mixed method design was employed to gather, analyse and report data (Table 1). Both quantitative and qualitative data were collected through an online survey and key informant interviews. Combining both data collection techniques allows better understanding of a research problem by converging broad numeric trends (quantitative) and detailed views (qualitative) data. Data are gathered concurrently and analyses are blended to create a more complete, in‐depth exploration of the research question [24].

**TABLE 1:** Mixed methods appraisal tool (MMAT), Version 2018.

Category of study designs	Methodological quality criteria	Responses			
		Yes	No	Cannot tell	Comments
Mixed methods	5.1. Is there an adequate rationale for using a mixed methods design to address the research question?	x			Case‐study methodology requires a mixed‐methods approach. The five aims of the study required a mixed method approach to gain a holistic understanding of the research topic.
	5.2. Are the different components of the study effectively integrated to answer the research question?	x			Each aim has been adequately addressed through the different components included in this study.
	5.3. Are the outputs of the integration of qualitative and quantitative components adequately interpreted?	x			The outputs are integrated through the identification of the barriers and enablers.
	5.4. Are divergences and inconsistencies between quantitative and qualitative results adequately addressed?	x			Two members of the research team independently reviewed three transcripts to ensure divergences and inconsistencies were discussed and resolved.
	5.5. Do the different components of the study adhere to the quality criteria of each tradition of the methods involved?	x			Identification of areas of trustworthiness has been discussed within the manuscript to ensure quality criteria has been met

A Project Advisory Committee (PAC) was established at the project outset, and members convened five times during the project. The PAC comprised Aboriginal and non‐Aboriginal representatives from the project team, ACSPs and community representatives. The aim of the PAC was to guide the project team and ensure relevance of activities and cultural safety.

Community consultation occurred from June through to September 2021. Members of the project team—including Author 5, who is an Aboriginal woman with Yawuru and Karajarri heritage and extensive networks in the Kimberley region—travelled throughout the Kimberley to hold in‐person interviews. This optimised participation of the range of ACSPs across the region.

Interviews were scheduled at a mutually suitable time and recorded for transcription purposes. Some interviews were also held virtually via Zoom or via phone.

Most participants completed the online survey prior to taking part in the interview phase, which allowed for continuity in results between both methods. At the request of some participants, the survey and interview were completed during the same meeting. This increased participation rates and ensured involvement of those participants who were unavailable to complete consultation activities at separate times.

### 3.3. Participants

Both purposive and snowball sampling were used to recruit ACSPs. Purposive sampling is a nonprobability sampling approach, whereby participants are selected who have the greatest amount of information and experience about the topic [25]. Snowball sampling is a chain‐referral method where initial participants recruit others from their network [26]. The purposive approach that seeks representativeness for each organisation was applied for this study [27]. A purposive sample representing managers and key aged care workers was identified within each stakeholder organisation, and individuals were contacted to be a part of the project. The application of multiple sampling methods and the inclusion of a range of stakeholders reduced data collection bias and improved participation rates.

The initial sample size included 10 key informant interviews and 30 survey respondents. Researchers contacted approximately 50 individuals from 13 ACSP operating in the Kimberley region at the time. The final study population comprised 16 representatives from 11 of these services.

### 3.4. Ethical Considerations

An introductory email was sent to management of ACSPs outlining the project and including a Project Information Sheet (PIS). A team member telephoned potential participants to explain the purpose of the project, why the service was being invited to participate and what their participation would involve, the risks and benefits and explain any part of the PIS needing clarification. All participants provided informed consent prior to the completion of the survey and interview. Ethics approval was obtained from the University of Notre Dame Australia (approval number 2020‐122F), Western Australian Aboriginal Health Ethics Committee (approval number RGS 1054) and Western Australia Country Health Services (approval number RGS 04287).

### 3.5. Instruments

#### 3.5.1. Online Survey

An electronic survey was distributed using QualtricsTM online survey software. Surveys were sent to management‐level representatives of all ACSPs to obtain high‐level data on service structure and delivery pre and post COVID‐19 restrictions. The survey comprised 22 questions. The first six were demographic questions (e.g., What is your role in the service/facility?). The remaining 16 questions asked participants about their experience providing care before and during the COVID‐19 restriction period, including nine allowing for open‐ended responses. The survey tool was first developed and reviewed by the research team and then sent to the PAC for review to establish preliminary evidence of content and face validity prior to implementation.

#### 3.5.2. Key Informant Interviews

Interviews were held with representatives of the ACSPs and lasted between 30 and 60 minutes. These were held via Zoom, telephone or in‐person, recorded and then transcribed for analysis. The interview schedule comprised seven questions relating to demographic information, how the service responded to changes and the experience of care provided during this period. The interview guide was created by experienced qualitative researchers in the project team and reviewed by PAC prior to implementation.

### 3.6. Data Processing and Analysis

Survey responses were entered into Microsoft Excel TM, and descriptive data were reviewed and reported. Due to the limited number of responses, only total counts and percentages were reported. Figures were created in Microsoft Excel TM. QSR NVivo 12 TM was used for data storage and management, coding and theme building for the interview transcripts. First, all audio‐recordings were reviewed and listened to by Author 1 to establish open codes. Individual transcriptions were coded line by line by Author 1 to ensure familiarity with the text. Braun and Clarks’ [28] six phase of thematic analysis was then used to identify patterns and themes within the transcripts. Lastly, the quantitative and qualitative results were integrated to identify key barriers and enablers associated with providing care over the COVID‐19 restriction period.

#### 3.6.1. Rigour

The rigour of this project was established through a second member of the project team independently coding three transcripts to establish member checking and to ensure credibility of the results. The main themes were discussed among both team members and differences debated until a consensus was reached.

## 4. Results

**FIGURE 1:**
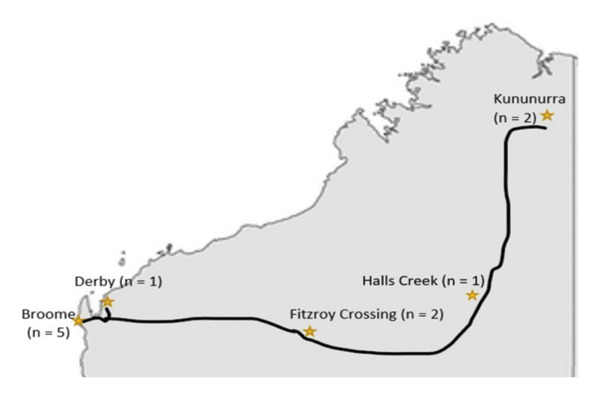
Demographic and geographical location of ACSPs who participated in this project.

A total of 16 participants from 11 ACSPs took part in the project. Figure 1 depicts towns serviced by the 11 ACSPs, with one providing amenities in both Broome and Kununurra.

### 4.1. Quantitative Results

A total of nine surveys were completed with six ACSPs (see Table 2).

**TABLE 2:** Role and service type of survey participants.

Service type	General role	Location
Community	Senior management	Derby
RACF	Senior management	Kununurra
Community	Medical staff	Broome and Kununurra
Community	Senior management	Fitzroy Crossing
Community	Outreach services	Fitzroy Crossing
Community	Outreach services	Yawuru community
Medical centre	Senior management	Halls Creek
RACF	Medical staff	Broome
RACF	Medical staff	Broome
RACF	Medical staff	Broome

Abbreviation: RACF, residential aged care facility.

Prior to the COVID‐19 outbreak, 13 services were provided across the nine ACSPs surveyed. The most common were in‐person care (66%) and home visits (55%). Four out of the nine ACSPs provided care to outreach services across the Shires of Broome and Derby. Eighty‐eight percent of ACSPs serviced between 20 and 59 clients per week, and only one ACSP serviced more than 99 clients over a one‐week period.

Care services changed for several ACSPs during the COVID‐19 restriction period. Facility and site activity, home visits and in‐person types of care decreased, whereas telephone and home visits increased. Some ACSPs noted additional requirements regarding screening and safety measures of patients and staff, fewer home visits and use of new communication strategies. These responses were common among care coordinators in Broome. Telehealth services increased for staff education and meetings. No changes occurred to remote consultation or monitoring. Broome and Kununurra regions reported a reduction in staff meetings.

An average of four staff worked within each ACSP. During the COVID‐19 restriction period, the length of care provided to clients changed for four out of the ten ACSPs. Of those four, care increased for three of these facilities and decreased for one. This may be the result of only having one person working for the service each week. Two ACSPs indicated care had decreased. The community development officer in Broome indicated that during COVID‐19 staff from other departments assisted them with providing care for the older people.

### 4.2. Qualitative Findings

A total of 14 transcripts were reviewed, comprising 12 one‐on‐one interviews and two group interviews (one with two participants and another with four participants (Table 3).

**TABLE 3:** Role and services of interview participants.

Service type	General role	Region
Medical service	Medical staff	Broome
RACF	Senior management	Kununurra
Medical service	Medical staff	Broome
^∗^Community and RACF		Kimberley region
Community	Medical staff	Kununurra and Broome
RACF	Medical staff	Halls Creek
^∗^Community	Senior management	Fitzroy Crossing
Community	Outreach services	Yawuru community
Community	Outreach services	Yawuru community
RACF	Medical staff	Broome
RACF	Medical staff	Broome
RACF	Medical staff	Broome
	Senior management	Halls Creek

Abbreviation: RACF, residential aged care facility.

^∗^Group interview.

Qualitative analysis uncovered three key themes and several subthemes in the consultation data. These are presented in Table 4 and described in detail thereafter.

**TABLE 4:** Qualitative themes.

Rapid changes to services	Importance of community	Long‐term health impacts
Changes in catchment area of service provision	Collaboration and sharing of resources	Physical health
Worry and fear of COVID‐19	Resident’s sense of place	Emotional health
Retention of services		Feelings of loneliness

#### 4.2.1. Rapid Changes to Services

A key theme of this project was ‘rapid changes to services’ during the COVID‐19 restriction period. All ACSPs quickly adapted service provision to ensure essential services continued to reach those in need. This required further planning and long hours to comply with mandatory government requirements regarding social distancing, cleaning, sanitising, temperature checks and use of personal protective equipment (PPE). Furthermore, many ACSPs had to adapt their processes for each community that they visited:

“So instead of bringing the oldies in Monday, Wednesday, Friday and having them altogether in the building, having their breakfast, doing the activities. We went in in the morning, cooked all the breakfasts up and delivered it out… Some of them closed the gates, some of them didn’t etc. So it wasn’t quite the same for every single one. So what we did was, we had effectively a designated person at the community gate that we could say, look here’s such and such breakfast, plop. Then they were quite happy to go in there. It stopped us going in, it stopped them coming out but they were still getting their meals which is obviously hugely important here. I mean food security is such a big thing. So they weren’t getting the activities, but they were still getting the service and were still getting the food.” (Senior management, Commonwealth Home Support Program, Fitzroy crossing)

Additional supports and supplies were often put in place to ensure the older people did not miss out on essential services. In some aged care facilities, nonessential staff were permitted limited site access unless for an emergency. Medical staff concerned for their patients’ safety, performed additional duties such as car park consultations, taking patients to hospital and personally delivering medications:

“So because they didn′t want any unnecessary visitors or unnecessary people, I would only be able to go in to see my patients if something had gone wrong, if they were acutely unwell. Then the nurse would contact me and say, please, can you visit?… So you had to rely on nurses keeping an eye out for deterioration of your patients.” (Medical staff, Broome Medical Clinic, Broome)

A subtheme was *‘changes in catchment area of service provision.*’ For community and outreach groups undertaking home visits, their services did not change; however, their catchment area increased as many Aboriginal elders went ‘bush’ and had their extended families returning home:

“So last year for COVID restriction, we had less residents because the protocol ‐ last year, I think we had four people or two people ‐ they got COVID‐19 infection in Broome. So we didn’t take any resident to come in. So that time, we have about 18 residents ‐ between 15 to 18 residents ‐ but after the lockdown, most of the people had COVID‐19 vaccine so we take in more residents.” (Senior management, Menkawum [Yuri Yungi aboriginal Medical Service], Halls Creek)

Many ACSPs commented on the *‘worry and fear’* that they, the older people and their families, felt around the uncertainty about COVID‐19. This led to increased educational sessions to try to ease their sense of worry. Based on these changes long‐term planning occurred to *‘retain services’* such as telehealth, working from home, car park consultations and assessing with basic health needs such a hygiene, a subtheme to the *‘rapid change in services.:*

“People were too scared to come in and we were pretty much being… told, unless it’s an emergency, stay at home and don’t come to the clinic…. No one could come in to relieve us and terribly for us at that time was that two nurses and a doctor or something in the nearby town… got COVID. So you can imagine the big scare… that it was being brought in by us.” (Medical staff, Silverchain, Broome)

#### 4.2.2. Importance of Community

Due to the impact of the changes generated through the COVID‐19 restriction period, the importance of community was a priority amongst ACSPs. Many noted a population influx in Aboriginal communities as people returned home, which increased the level of care ASCP were providing to remote communities:

“…All our clients are Indigenous. So for us, it’s living as well as possible on country with family and community. That’s what we are always aiming for. We can’t always achieve it because if somebody gets too sick or too demented or whatever then it may mean going to a frail aged facility off country. We do as much as we can.” (Kimberly Aged and Community Services Focus group, West Kimberly)

*‘Organisational resources and working together’* was a subtheme to the importance of community. Many services were working together for the first time to support older people and their wider community, which included the support and collaboration with the local council. Each organisation developed specific procedures to help each other manage the increasing workloads due to the COVID‐19 restrictions:

“…when I had the first meeting with the hospital, I said to them that we’re going to work together and this is our strategy. You look after the clinical side because there was a lot of changes that had to be made to the hospital. I said, let us look after the health promotion side and the communities. We’ll deal with the 45 communities because people came back from … and we went house‐to‐house.” (Senior management, Commonwealth Home Support Program, [Nindilingarri Cultural Health], CEO, Fitzroy Crossing).

*‘Residents’ sense of place’* emerged as a subtheme as many ACSPs valued and respected the importance the older people placed on country and their sense of community. ACSPs performed additional checks to monitor who was coming in and out of their service and used creative measures to make sure the community was aware of these changes. Many ACSPs saw their patients’ familial connections and cultural traditions impacted and empathised with them as many chose to be with their families and risk contracting COVID‐19:

“Actually the elders that did felt it very impacted on their health because they would love to go on country and get out in fresh air, but because everything was locked down they were actually like suffocating in their own homes.” (Senior management, Nyamba Buru Yawuru, Yawuru community)

#### 4.2.3. Long‐Term Impacts

Many ACSPs had to modify services to protect the health of the older people, which often significantly delayed treatment for some patients. This has had long‐term impacts on many patients physical and emotional health. *‘Physical health’* was impacted as checkups for comorbid conditions were often cancelled to limit travel to remote communities. For example, podiatric services were placed on hold during lock down, which led to long‐term effects:

“We actually stopped, for quite a while there, we stopped things like the dietician, the podiatrist, the diabetes educator, they didn’t go out because we didn’t see them as essential services during that initial lockdown. Now, once we got to the two‐month mark and people hadn’t had their feet looked at for two months, then they became a bit more important and we started letting them back in. Yeah, it was a case of we still maintained our basic services in our clinics and they were quite busy because they were testing as well, so testing for COVID out there as well. What people could access was slightly less and it was mainly to do with the Allied Health side of things.” (Medical staff, Kimberly Aged Care & Community Services, Broome)

*‘Emotional health’* became of great concern as social activities and visits from family members were limited. This increased the level of stress experienced by the older people as well as those providing care:

“It definitely impacted their emotional health, I think, because of lockdown. When WA was having a few COVID cases, residential facilities were on lockdown. Families were not allowed to come in for a couple of days. There was a communication breakdown. They were worried about the family, vice versa on both sides.” (Medical staff, Sliverchain, Broome)

‘Loneliness’ was another aspect associated with older persons’ emotional health. Many commented that the older people were more concerned about losing contact with their family than a decline in their health:

“Do you know what, our residents here, it would be awful, but I think they would rather catch a cold than be lonely, which sounds horrible” (Medical staff, Southern Cross, Germanus Kent House, Broome)

## 5. Synthesis of Quantitative and Qualitative Findings

### 5.1. Barriers and Enablers

Barriers and enablers were both identified in the quantitative and qualitative findings and are synthesised in Table 5. Several barriers arose that limited care access/service access restrictions in resources and equipment and staffing shortages. ACSPs circumvented these barriers by adapting services quickly, responding to shortages in supplies and focussing on providing additional training and supporting staff.

**TABLE 5:** Barriers vs. enablers.

Barriers	vs.	Enablers
Access to services	vs.	Adapting existing services
Access to additional services became difficult during the COVID‐19 restriction period. This included additional services such as hospitals and pharmacies. Many ACSPs provided door‐to‐door services, so patients did not have to leave their community.		

Resourcing and equipment	vs.	Supply of equipment and resources
Resourcing and medical equipment were in short supply when the COVID‐19 restriction period first occurred. However, this was overcome very quickly and now backup supplies are available for many services.		

Staffing shortage and burnout	vs.	Staff training and support
Staffing shortages, lack of cultural understanding and skilled workers, working to fill other roles and long employment processes were of great concern to ACSPs. Many organisations focused on staff training, upskilling and providing additional support, which seemed to help the heavy workloads.		

Government mandates, reporting and funding	vs.	Changes strategies and planning procedures
Key information recommended by organisations’ head offices, often based in Perth, often conflicted with what was occurring the Kimberly region. There was also stress around funding and government reporting. On reflection, ACSPs felt better about future crisis as strategies and procedures were now in place.		

## 6. Discussion

Using the Kimberley region as a case study, this project sought to explore experiences shared and strategies employed by ACSPs delivering care during a global health crisis. Several key themes emerged during consultation: rapid changes to services, importance of community, and long‐term impact on the older population.

Themes described above were impacted by several operational, personal, policy and workforce barriers (i.e., access to services, resourcing and equipment, staffing shortages and burnout, and government mandates, reporting and funding). Although some barriers may be the result of the remoteness of the Kimberly, as the level of remoteness often inhibits the health services delivery [11, 12], these were impacted significantly more due to COVID‐19 mandated restrictions. For example, some ACSPs had to complete mandatory reporting that required travelling long distances. This took them away from their daily role and service provision.

Enablers identified to overcome barriers included adapting existing services, supply of equipment and resources, staff training and support and changes to strategies and planning procedures. Creative use of telehealth services helped maintain and enhance service efficacy during the pandemic. Latifi et al. [18] similarly found COVID‐19 lead to the rapid onset of telehealth services that were used for the first time by many medial health professionals. A scoping review focussing on the use of telehealth during the COVID‐19 pandemic found that 84.9% of the articles reviewed celebrated the use of telehealth [29]. The utilisation of telehealth during the COVID‐19 pandemic has been described providing a sense of control and safety and efficacity for medical procedures [30], with a more broader application expanding to improving the health outcomes of those from different racial, age and socioeconomic backgrounds [31].

The use of telehealth in a palliative home care setting from Steindal et al.’s [32] scoping review indicated that telehealth was easy and effortless, the visual features enhanced communication and care, symptom management and self‐management were promoted by telehealth and perceptions of improved palliative care at home were reported. Dai et al. [33] conducted a retrospective cohort analysis of telehealth utilisation in RACFs in 2020 for Victoria and New South Wales and found that 93% of general practitioner consultations were conducted over the telephone, with pension holders and those residing in rural areas more likely to use the telephone option compared with a virtual video call. Similarly, for this study based in WA, the complexity in the use of telehealth arose in trying to implement this technology in a location where the infrastructure was limited. Educational sessions and home visits helped to overcome some barriers. Added constraints due to the pandemic lead to many services struggling to meet community and staff needs. However, ACSPs that provided further training and held regular staff meetings were able to adapt to the changing landscape and restrictions. Thorburn et al. [13] also found that timely and well‐designed strategies employed at the start of COVID‐19 kept Kimberley communities safe.

Since the COVID‐19 pandemic, healthcare services have shifted and continue to provide telehealth and virtual healthcare interactions. The addition of technology has shifted the in‐person mode of care to an online and/or hybrid model [34] and continues to be an effective and affordable health care approach [17]. Further work in the nursing sector has refined the competencies for telehealth practices by advancing practice, improving effectiveness and developing curriculum within education settings to improve the delivery of telemedicine [35], while implications for telehealth postpandemic has resulted in changes in payment methods, privacy and licencing regulations [36]. Overall, the use of telehealth was a welcomed addition to the traditional models of care offered during the COVID‐19 restriction period found for this study. However, further considerations for older people and the use telehealth may need to be considered. For example, Dai [37] found hearing, vison or cognitive impairment and their ability to use technology limiting factors for the use of telehealth. Additional infrastructure to support the use of telehealth postpandemic is required to support the continuation of this model of care in a remote setting.

A Stakeholder Conversation was held in Broome on 18 August 2022, during which study finding were presented and discussed. The majority of Kimberley ACSPs were represented at this meeting. Copies of the final project report [38] were also disseminated to stakeholders and members of the PAC.

## 7. Strengths and Limitations

A moratorium on research activities enacted by the Kimberley Aboriginal Health Planning Forum (KAHPF) Research Subcommittee to protect ‘…Aboriginal health and wellbeing in the face of this unprecedented global health threat’ [39] initially delayed the project. Once approved, the project commenced but interviews via videoconferencing, telephone or in person if possible and agreed upon by the participant.

The project was limited by the participation size. Service staff were time‐poor, under continuous pressure and consumed with the immediate COVID‐19 health emergency. As a result, many were simply ‘too busy’ or unwilling to participate in the study. Personal connections and rapport between members of the project team and community/agencies encouraged individuals to participate. Others opted to complete either the survey or the interview or to complete these simultaneously to save time. Given the low participation rates, the team elected to eliminate focus group/yarning circle stage of consultation and used data gathered from interviews and surveys only. Nonetheless, the quality of the data collected was of rigorously sound quality.

## 8. Conclusions and Recommendations

Unexpectedness of pandemics such as COVID‐19 means it is imperative to develop strategies that work effectively across sectors and ACSPs in the future. Findings of this project can inform decision‐making processes and encourage stronger links between those providing aged care. It may also assist in preparing for potential pandemics and health crises amongst this and similar populations throughout remote Australia. Specific recommendations are as follows:

1. Improve access to remote communities and enhance infrastructure and telehealth methods to build capacity for these services in health emergencies.
2. Increase the number of culturally trained and skilled staff.
3. Provide tailored recommendations for regions and areas rather than a ‘one size fits all’ approach to delivery of services in health emergencies.
4. Increase the availability of appropriate funding to support aged care services during health emergencies.
5. Incorporate plans earlier to better assess situations and develop strategies to reduce lockdown measures and protect RACFs residents and staff.
6. Provide COVID‐19 specific funding opportunities to allow for more staff to provide support during a pandemic period.

These findings and recommendations may contribute to a better understanding of successful strategies in providing care to older Kimberley residents, in particular Aboriginal people. By providing important insight into staff experiences, the project may also inform decisions regarding support for front line workers during similar health emergencies. The project highlights the need to improve how aged care workers are supported during disaster scenarios as this, above much else, leads to improved outcomes in health and wellbeing for older members of any community.

## Author Contributions

Amanda Timler: methodology (equal), project administration (equal), resources (supporting), validation (supporting), visualisation (supporting), writing–original draft preparation (leading) and writing–review and editing (leading). Jane Gaspar: conceptualisation (supporting), data curation (lead), formal analysis (equal), investigation (supporting), methodology (supporting), project administration (equal), validation (equal), writing–original draft preparation (equal) and writing–review and editing (equal). Karen Clark‐Burg: conceptualisation (lead), methodology (supporting), project administration (equal), investigation (lead), resources (supporting), supervision (supporting) and writing–review and editing (supporting). Caroline Bulsara: conceptualisation (leading), methodology (equal), project administration (equal), investigation (lead), resources (supporting), supervision (supporting) and writing–review and editing (supporting). Maria Morgan: conceptualisation (supporting), methodology (supporting), supervision (supporting) and writing–review and editing (supporting). Cathryn Josif: conceptualisation (supporting), methodology (supporting), project administration (supporting), resources (supporting), supervision (supporting) and writing–review and editing (supporting). Erina Myers: conceptualisation (supporting), data curation (supporting), formal analysis (equal), methodology (equal), project administration (equal), resources (supporting), validation (supporting), visualisation (supporting) and writing–original draft preparation (supporting).

## Funding

This project was supported by funding from the Department of Jobs, Tourism, Science, and Innovation, Government of Western Australia grant. Open access publishing was facilitated by the University of Notre Dame Australia, as part of the Wiley‐The University of Notre Dame Australia agreement via the Council of Australasian University Librarians.

## Disclosure

An introductory email was sent to management of the selected ACSPs outlining the project and including a Project Information Sheet (PIS). A team member telephoned potential participants to explain the purpose of the project, why the service was invited to participate and what their participation would involve, the risks and benefits, and explain any part of the PIS needing clarification.

## Ethics Statement

Ethics approval was obtained from the University of Notre Dame Australia (approval number 2020‐122F), Western Australian Aboriginal Health Ethics Committee (approval number RGS 1054) and Western Australia Country Health Services (approval number RGS 04287).

## Consent

All participants provided informed consent prior to the completion of the survey and interview.

## Conflicts of Interest

AT, JG and EM received financial support and MM, CJ, AT and KCB received conference support through the Department of Jobs, Tourism, Science, and Innovation, Government of Western Australia grant.

## Endnotes

^1^Aged care can be defined as assistance provided to older individuals in their homes or residential facilities, which includes healthcare, daily living and accommodation support 3. Australian Government Department of Health and Aged Care. What is aged care? 2026 [Available from: https://www.health.gov.au/health-topics/aged-care/about-aged-care/what-is-aged-care.

^2^Telehealth refers to the use of information and communication technologies to deliver diagnosis, treatment, preventive, and curative aspects of healthcare services to care recipients and their care providers 15. Australian Government Department of Health. Telehealth 2025 [Available from: https://www1.health.gov.au/internet/main/publishing.nsf/Content/e-health-telehealth, 16. World Health Organisation. Global strategy on digital health 2020‐2025. 2021.

## Data Availability

The data that support the findings of this study are available on request from the corresponding author. The data are not publicly available due to privacy or ethical restrictions.
